# Indirect approach for estimation of forest degradation in non-intact dry forest: modelling biomass loss with Tweedie distributions

**DOI:** 10.1186/s13021-016-0051-z

**Published:** 2016-06-29

**Authors:** Klaus Dons, Sushma Bhattarai, Henrik Meilby, Carsten Smith-Hall, Toke Emil Panduro

**Affiliations:** 1 Informi GIS, Stationsparken 37, 2600 Glostrup, Denmark; 2 Faculty of Science, University of Copenhagen, Rolighedsvej 23, DK-1958 Frb C, Copenhagen, Denmark; 3 United Nations Development Programme, UN House, Pulchowk, GPO Box 107, Lalitpur, Kathmandu, Nepal

**Keywords:** Compound Poisson distribution, Spatial analysis, REDD+, Forest monitoring, Tanzania

## Abstract

**Background:**

Implementation of REDD+ requires measurement and monitoring of carbon emissions from forest degradation in developing countries. Dry forests cover about 40 % of the total tropical forest area, are home to large populations, and hence often display high disturbance levels. They are susceptible to gradual but persistent degradation and monitoring needs to be low cost due to the low potential benefit from carbon accumulation per unit area. Indirect remote sensing approaches may provide estimates of subsistence wood extraction, but sampling of biomass loss produces zero-inflated continuous data that challenges conventional statistical approaches. We introduce the use of Tweedie Compound Poisson distributions from the exponential dispersion family with Generalized Linear Models (CPGLM) to predict biomass loss as a function of distance to nearest settlement in two forest areas in Tanzania.

**Results:**

We found that distance to nearest settlement is a valid proxy variable for prediction of biomass loss from fuelwood collection (p < 0.001) and total subsistence wood extraction (p < 0.01). Biomass loss from commercial charcoal production did not follow a spatial pattern related to settlements.

**Conclusions:**

Distance to nearest settlement seems promising as proxy variable for estimation of subsistence wood extraction in dry forests in Tanzania. Tweedie GLM provided valid parameters from the over-dispersed continuous biomass loss data with exact zeroes, and observations with zero biomass loss were successfully included in the model parameters.

## Background

Measuring forest degradation using remote sensing (RS) is generally more challenging than measuring deforestation [1] and, combined with scarce funds and insufficient technical capacity in many developing countries, lack of estimates of emissions from forest degradation is limiting implementation of reduced emissions from deforestation and degradation (REDD+) [2]. The indirect RS approach that maps the infrastructure facilitating degradation, such as settlements and roads, offers a way to delineate intact forest from non-intact forest and to model and estimate emissions from forest degradation in non-intact forest [3]. This approach may also be used to estimate past carbon emissions from present measurements of degradation activities and establishing the historic baseline necessary to demonstrate additionality [4]. Most forest degradation studies on carbon stock changes focus on degradation in undisturbed humid forests with high levels of carbon stock per hectare [3, 5, 6]. However, it has been suggested that degradation in dry forests may be more widespread on a global scale [7] and that such degradation needs to be quantified [8, 9]. Degradation in dry forests is likely to imply a slow reduction in carbon stock over time [8], including subsistence extraction due to underlying drivers such as population growth and poverty [10] which further limits the use of RS for estimation of emission levels. The potential carbon benefit per unit area in dry forests is low, but countries may access benefits from REDD+ funding if they can quantitatively monitor significant degradation activities over large forest areas likely to be degraded [4]. Forest degradation can be defined at national or sub-national levels, as can the specific degradation activities targeted for monitoring [7]. In general, degradation drivers can be categorized as either subsistence wood extraction activities, commercial activities, or uncontrolled wild fires [4, 11]. The first two categories are compatible with von Thünen’s model of agricultural land use, saying that harvesters are willing to travel longer for high value products [12]—low value subsistence products are extracted in proximity to population centres, whereas higher value commercial products can be harvested in remote forest areas. In consequence, we would expect that the level of subsistence degradation decreases with increasing distance to populated areas [13, 14].

In this study, we use an indirect RS approach to estimate biomass loss, and hence carbon emissions, in a tropical dry forest. It is applied to estimate biomass loss from both commercial and subsistence activities. A common problem in inventories is that sampling of biomass loss produces continuous data including a high number of true zeroes. We define true zeroes as those that represent actual response outcomes, i.e. ‘no disturbance’ in a particular sampling unit and not zeroes created by missing data. Statistical data analysis of such zero-inflated continuous data is challenging as ordinary distributions, such as normal or gamma, fit the data poorly and log-transformations or similar are not possible due to the zeroes [15]. A common solution in studies of biological resources, such as in marine biology and fisheries, has been to sub-set or transform the data with a constant to remove the zeroes and then use standard models such as generalized linear models (GLM) with log-normal or gamma distributions on the remaining continuous data [16, 17]. However, the assumptions of these approaches are associated with statistical problems [18, 19] and have been found to overestimate the quantity of biomass from fisheries [20]. Another solution is to use a separate model for the zeroes, e.g. delta-type models by use of logit, or probit to estimate the rate of zeroes, followed by a model of the continuous positive data [21]. Such two-step models allow different estimates for the two components and while this has been found useful in econometric studies [15] it is less applicable to biomass data because of limitations to application in a multiplicative structure [19]. Hence, when modelling forest degradation, the zeroes are an inherent part of the data and should be actively included for correct parameter estimates of biomass loss. Here we apply the exponential dispersion model (EDM) family of distributions [22, 23]. EDM distributions are response distributions for GLM and include the Tweedie family of distributions, which has proven especially useful for modelling positive continuous data with a proportion of exact zeroes [24, 25]. Tweedie GLM implements a multiplicative structure on the dependent variable [19] by combining the discrete and continuous probabilities and thereby provides valid estimates where true zeroes are included in the estimate of the continuous response variable. The implementation in the established GLM framework makes model results comprehensible to readers. EDMs have been available for decades but have a density function which is analytically intractable and thus not included in commercial statistical software until recently [26].

This paper assesses the accuracy of an indirect RS approach to estimate commercial and subsistence wood extraction in dry tropical forests. We use Tweedie Compound Poisson distributions from the exponential dispersion family with GLM (CPGLM) to predict biomass loss as a function of distance to nearest settlement. Furthermore, we demonstrate a simple GIS approach to establish area-based CPGLM predictions of biomass loss from subsistence degradation activities for potential application to REDD+ monitoring systems.

## Results

The average biomass loss per hectare and the number of extraction incidents per hectare are presented for the degradation categories in Table 1. Main categories are commercial charcoal and total subsistence extraction, whereas fuelwood is a sub-group that is also included in total subsistence. Considering the descriptive data, charcoal production is the most severe degradation activity with more than 3  Mg per ha. An interesting finding is that trees that are cut for charcoal contain on average four times more biomass than trees for subsistence. Average biomass loss per subsistence extraction incident is 40.4 kg compared to 186.4 kg per tree used for charcoal. The estimates are averaged across the total sampled area at each of the study sites, but are not spatially stratified within each study area.

**Table 1:** Observed levels of wood extraction

	Biomass loss (Mg ha^−1^)			Number of stumps ha^−1^		
	IDO	KIW	Total	IDO	KIW	Total
Fuelwood	1.226	1.144	1.182	43	28	35
Total subsistence (incl. fuelwood)	3.425	2.678	3.027	97	56	75
Commercial charcoal	2.102	5.508	3.915	9	30	21
Total	5.527	8.187	6.942	106	86	96

Descriptive data on categories of wood extraction measured in 62 sample plots: 29 in Idodi ward (IDO) and 33 in Kiwele ward (KIW)

Parameter estimates for the CPGLM are presented in Table 2. The effect of location was tested on all models but only found significant in the model predicting fuelwood. However, ANOVA tests with a global model without location showed no significant improvement to model fit. Both models are shown in Table 2 though only global models are included and discussed henceforth. Parameter estimates for the global models are significant at level p < 0.01 and have negative spatial trend (Slope β). The parameter estimates for fuelwood and total subsistence use show that biomass loss decreases with increasing distance to nearest settlement. The slope parameter for charcoal is non-significant.

**Table 2:** Parameters for the models of biomass loss

Dependent variable	*P*	*ϕ*	Intercept *α*	Location effect γ (KIW = 1)	Spatial trend (slope *β) *
Fuelwood	1.5579	23.1135	6.48168 (0.61441)***	1.11205 (0.53172)*	−0.00098 (0.00022)***
Fuelwood (global)	1.5627	24.0375	6.45821 (0.65354)***	–	−0.00074 (0.00022)**
Total subsistence ext	1.5471	22.7754	6.48894 (0.47176)***	–	−0.00038 (0.00014)**
Charcoal	1.4475	142.0440	6.38165 (0.75172)***	–	−0.00024 (0.00022) *ns*

Parameters of CPGLM models (Eq. 4) for prediction of biomass loss as a function of distance to nearest settlement (meters) with density function *P* dispersion *ϕ*. For fuelwood a model with location dummy [Kiwele ward (KIW) = 1] and a global model are included. The models are described under the Data analysis paragraph in the “Methods” section

*Significance levels* p > 0.1 ‘ns’, p < 0.1, * p < 0.05, ** p < 0.01, *** p < 0.001

**Fig. 1:**
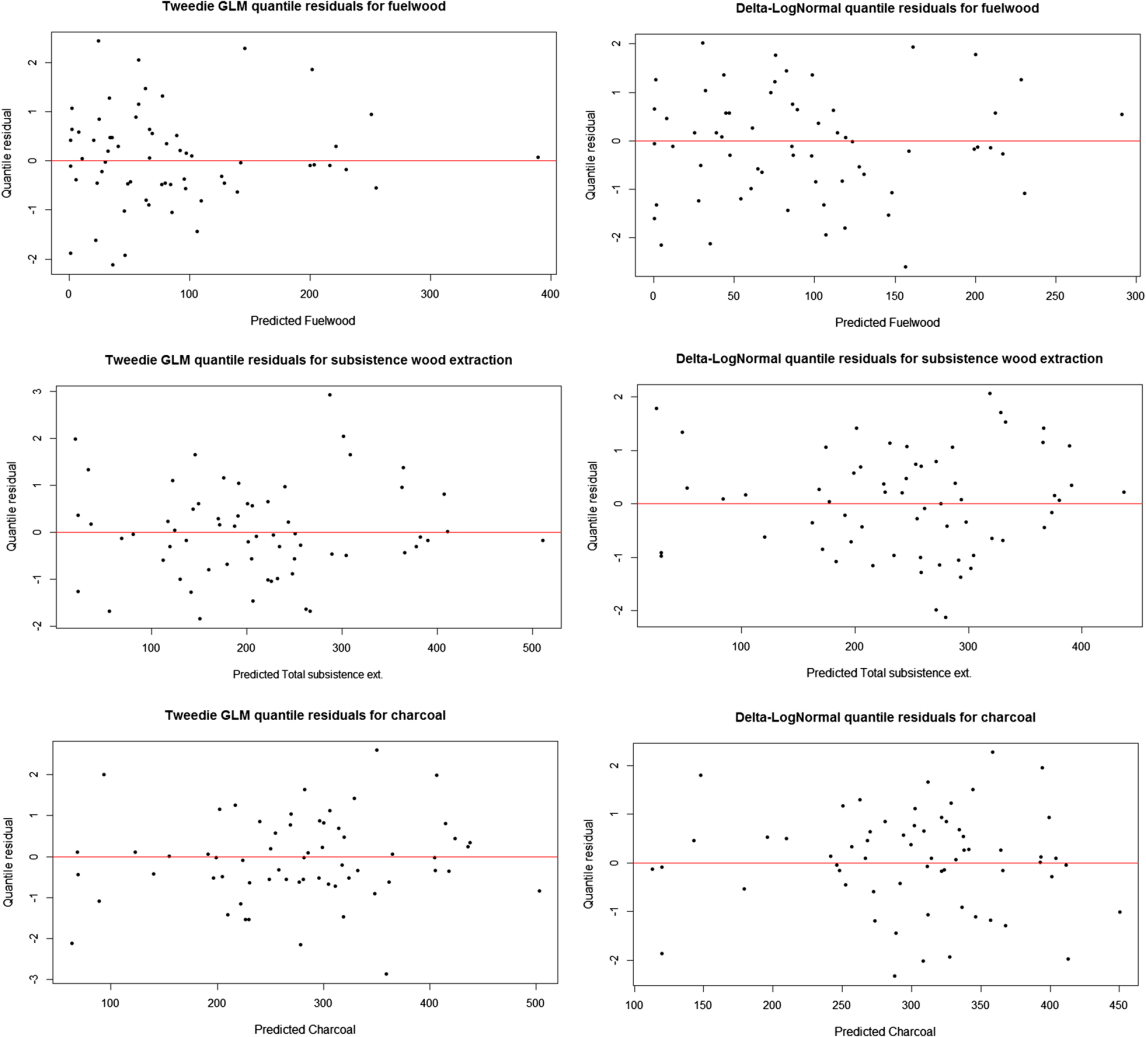
Quantile residuals of Tweedie GLM (*left*) and delta log-normal models (*right*). Predicted values of wood extraction by distance to settlement against quantile residuals. (*Upper*): Fuelwood, (*middle*): Total subsistence wood extraction, and (*lower*): Charcoal

**Fig. 2:**
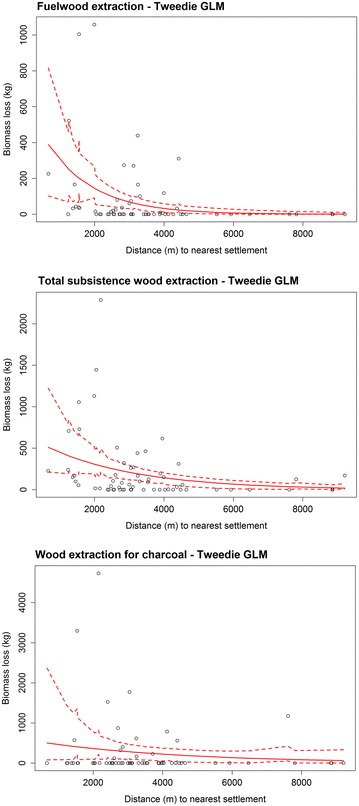
Biomass loss models by type of wood extraction. Biomass loss by wood harvested for fuelwood (*upper*), total subsistence (*middle*), and charcoal (*lower*) plotted against distance to nearest settlement. Model prediction by Tweedie GLM (*solid red line*) with 95 % confidence intervals by Jackknife bootstrapping (*dashed red lines*)

The application of CPGLM is a novel approach for prediction of forest biomass loss. Therefore, we compare the performance of CPGLM to corresponding delta log-normal models. The AIC of the CPGLM models fuelwood, total subsistence wood extraction and charcoal are: 412.2, 618.6 and 312.2, respectively. The delta log-normal models have similar AIC levels: 410.2, 621.2 and 317.1. Residual plots are provided in Fig. 1 to demonstrate that the fit to the non-transformed data including zeroes is reasonable and comparable to the residuals of the corresponding delta log-normal models. The residual plots show acceptable model fit and model parameters may therefore be applied for prediction of biomass loss by subsistence use. Fig. 2 illustrates CPGLM predictions, including 95 % confidence intervals, compared with measured values of biomass loss in the field. Highest uncertainty on the prediction of biomass loss is observed within approximately two kilometres from the forest edge.

### Application of CPGLM for spatial prediction of biomass loss

**Fig. 3:**
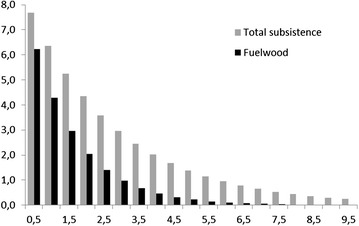
Biomass loss and distance to nearest settlement. Biomass loss (Mg ha^−1^) from wood harvest for fuel and total subsistence at increasing distance intervals from nearest settlement (km)

**Fig. 4:**
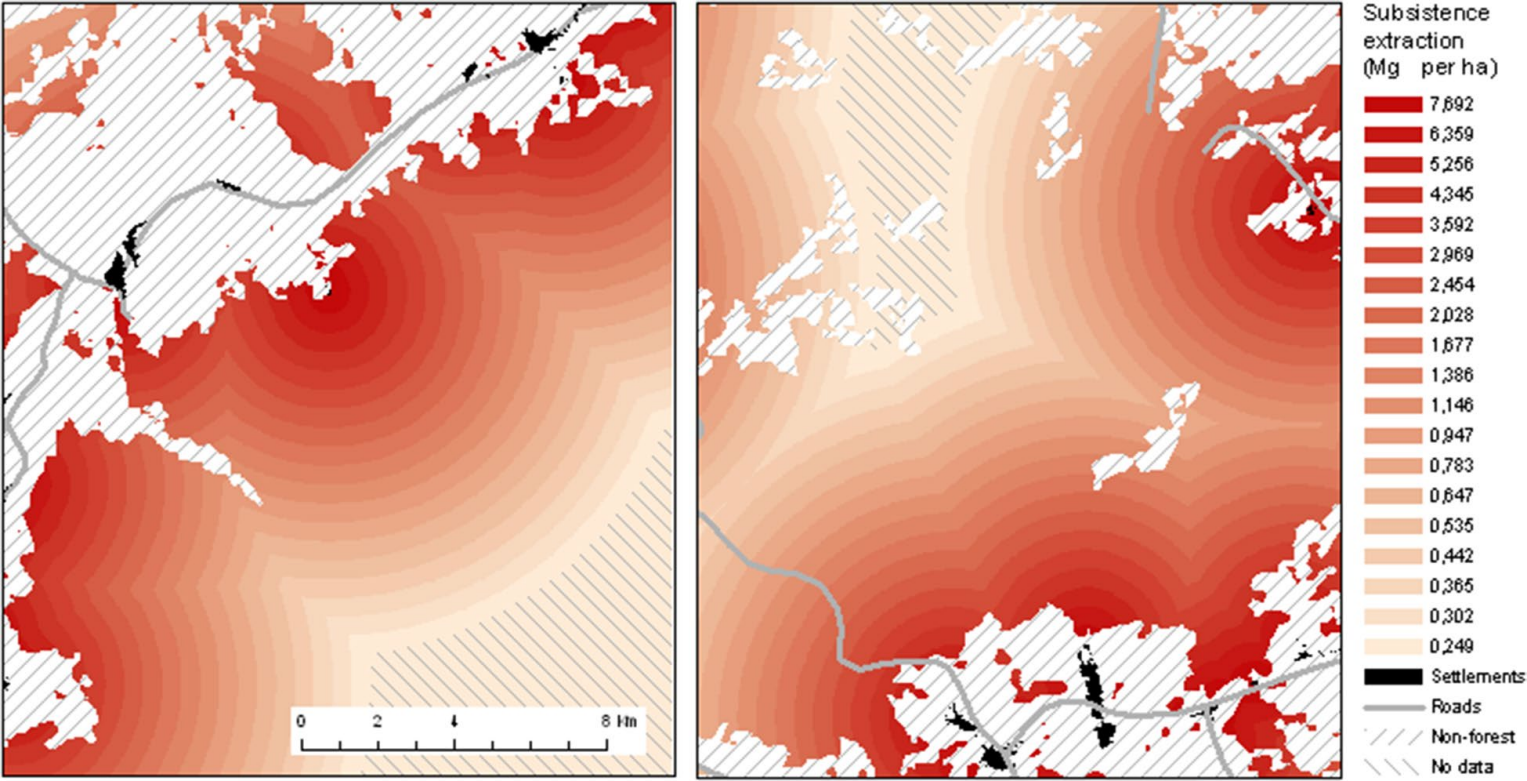
Biomass loss across the study area. CPGLM-based prediction of biomass loss by total subsistence wood extraction applied to forests in the study areas using multiple ringbuffer vectors at 100 m intervals

In acceptance of CPGLM model fit and significance of parameter estimates we put the model to work and apply location-based average biomass loss rather than total averages per forest as presented in Table 1. Total average subsistence wood extraction was about 3 Mg ha^−1^. Figure 3 ilustrates how CPGLM predicted levels of subsistence wood extraction declines with increasing distance. Biomass loss is above average within 3 km from settlements where it drops to below average. Our data extends to approximately 9000 m, but it seems that total subsistence harvest continues beyond our transects. Predicted biomass loss in Fig. 3 are taken from CPGLM at 100 m intervals. These are projected to area measurements by simple vector features containing concentric buffer rings at 100 m distances (Fig. 4). The 100 m interval applied here is for illustrative purposes. The use of vector GIS for projection of CPGLM predictions allows for any chosen interval though computational power limits very high spatial resolutions. The decrease in biomass loss by total subsistence wood extraction with distance from nearest settlement is evident in Fig. 4. The map shows only extraction levels in forest areas within 9500 meters from a settlement. Areas beyond this distance and non-forest areas are hatched out.

## Discussion

We applied a CPGLM model for estimation of biomass loss by subsistence wood extraction in non-intact forest areas as a function of distance to nearest settlement. Model parameters demonstrated significant decrease in biomass loss with increasing distance to nearest settlement. The result was valid for fuelwood (p < 0.001) and for total subsistence wood harvest (p < 0.01). The per plot levels of wood extraction for charcoal production were higher than that of subsistence harvest. All charcoal extraction sites but one were situated within 4500 m from nearest settlements, but otherwise charcoal extraction does not seem to follow a specific spatial pattern with regard to settlements. This was expected considering von Thünen’s theory of locational rent. From field studies we experienced that charcoal producers’ choice of extraction site follows the availability of a combination of tree species and ground surface conditions. The high value of the product makes the transportation cost worthwhile and we suggest that commercial degradation activities are perhaps best detected with direct RS approaches focusing on canopy cover changes. Because of the likely acceptance of longer travel distances [27] indirect indicators, such as infrastructure and concentration of population, may not apply for estimation of charcoal extraction.

In the case of sampling for biomass loss, most models applied are not able to facilitate a multiplicative structure allowing for the zeroes to take part in the final prediction of biomass. CPGLM seems to provide good fit for this type of data, so why has CPGLM not already been widely applied for estimation of forest degradation? One reason may be that Tweedie models in general have been seen as intractable and perhaps inaccurate as most common optimization procedures previously available are inappropriate [26]. In later years, improvements in optimization routines and statistical software [28] have increased the use of the models [26]. In recent ecological studies, CPGLM has been found particularly useful for estimating biomass from fisheries with no-catch occasions and the CPGLM seems to provide more accurate estimates and better fit to these data than, e.g. delta approaches [18, 19, 29, 30]. The Tweedie CPGLM has also been applied with success for near-optimal modelling of monthly rainfall [24]. A few studies on terrestrial ecology has also applied CPGLM to model for example reproductive capacity of moss [31] and the area of forest fires [32].

We also found good fits with the CPGLM and it was possible to provide highly significant predictions of biomass loss from subsistence wood harvesting as a function of increasing distance to nearest settlement. Model performance was comparable with delta log-normal models in terms of AIC and quantile residuals, but an advantage of CPGLM is that the response variable is maintained at its original scale, thereby providing higher variance stability compared to models that need back-transformation of the dependent variable in order to derive predicted values at original scale [20]. The predictions were used to produce maps of forest degradation in order to provide full area-based predictions of subsistence biomass loss. The use of concentric buffer distances with average extraction levels predicted by the CPGLM model for different distance intervals is simple and easy to implement at various spatial resolutions. It requires a forest vector, e.g. from Landsat, and settlements from remote sensing or existing GIS. This indirect remote sensing based prediction of subsistence wood extraction can be applied across large geographical areas using default values for extraction-distance relationships or be locally calibrated depending on the chosen accuracy level for measurement of subsistence extraction under REDD+.

In spite of the optimism regarding the model fit by CPGLM, this study includes a number of limitations. Biomass loss is estimated through two models. A stem shape model for conversion of stump DM to DBH and an allometric model for DBH to biomass (kg per tree). Although stumps represent true evidence of wood extraction, the use of two models introduces unquantifiable uncertainty in the biomass loss estimates. The spatial analysis tool illustrated here includes only few variables. Proxy variables such as population pressure, access roads, cost-distance and forest productivity (normalized difference vegetation index) were not included. It is expected that a larger study with more field sample plots would gain improved model fit by including more relevant proxy variables in a multiplicative way, e.g. by establishing various spatial predictions individually for a multiplicative raster surface overlay. Another improvement to this study would be to investigate the possible effects of spatial autocorrelation as villages and plots follow similar spatial distributions. This could lead to similarities in the data that may be confounded with the wood harvest. Finally, in this study we have assumed that harvested wood equals biomass loss. In doing so we have not included the potentially increased productivity in remaining trees that may result from reduced competition.

CPGLM offers potential for REDD+ monitoring approaches as we may expect better model fit for zero-inflated continuous data, thereby reducing the chance of overestimating biomass loss. Monitoring of forest degradation with remote sensing has been viewed as more complex and challenging than for deforestation. The introduction of degradation into REDD+ at COP 13 in Bali [33] was recently described as the end of the idea of a simple forest-based climate change mitigation system [34]. However, in this study we suggest a way to simplify quantification of subsistence wood extraction which has been identified as the most complex activity to estimate by remote sensing [4, 27].

If integrated in a GIS environment and locally calibrated, accurate estimates of subsistence wood harvest levels may be expected from non-intact dry forest areas using CPGLM, although further studies are needed to demonstrate applicability outside of the study areas assessed here.

## Conclusions

We assessed the accuracy of an indirect remote sensing approach to estimate commercial and subsistence wood extraction in dry tropical forests in Tanzania. We used Tweedie Compound Poisson distributions from the exponential dispersion family with GLM (CPGLM) to predict biomass loss as a function of distance to nearest settlement. We found that levels of fuelwood extraction as well as total subsistence wood harvest decrease significantly with increasing distance to nearest settlement. The level of biomass loss associated with commercial charcoal production does not follow a systematic spatial pattern related to settlements in the study areas. CPGLM offers potential for REDD+ monitoring approaches as we expect better model fit for continuous data with high numbers of true zeroes, thereby reducing the chance of overestimating biomass loss. Based on the present results we suggest a low cost GIS approach to establish area based CPGLM predictions of biomass loss from subsistence wood extraction using distance to nearest settlement as proxy variable. Further studies are needed to demonstrate if the approach is valid on a regional level for implementation in REDD+ monitoring systems.

## Methods

### Study area

**Fig. 5:**
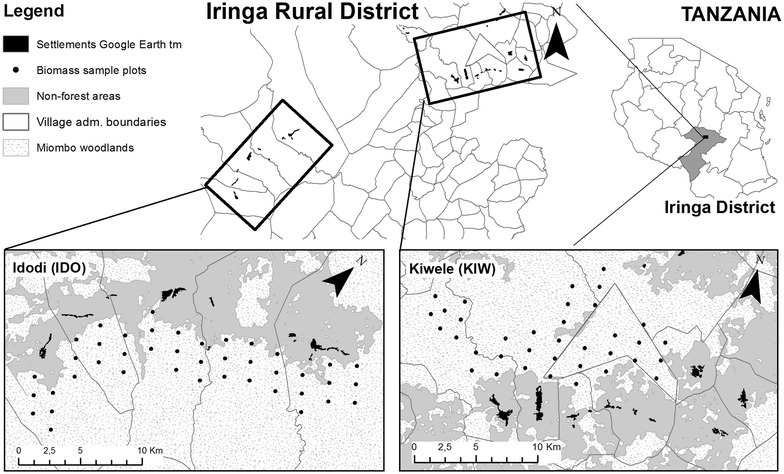
Overview of study areas in Iringa Rural District in Tanzania. (*Upper right*): an overview map of Tanzania and the location of Iringa District, (*upper middle*): Iringa Rural District with delineation of the two study sites. (*Lower left*): The Idodi site including village administrative boundaries, settlements digitized from Google Earth™, forest and field plots. (*Lower right*): The Kiwele site

A third of Tanzania’s forested area is covered by Miombo woodlands [35] that are widely used for fuelwood collection and charcoal production while timber extraction is less common [36], likely due to a low density of commercially valuable timber species. In Iringa Rural District, we selected two study sites with settlements distributed along the Miombo forest edge (Fig. 5). The south-western site (35°2′–35°14′E and 7°46′–7°58′ S) is located in and around Idodi ward (IDO), about 100–150 km from Iringa town, including the villages of Makifu, Mahuninga, Tungamalenga, Idodi, and Mapogoro. The second site (35°31′–35°42′E and 7°30′–7°37′S) is in Kiwele ward (KIW) 45 km north-west from Iringa town and includes the villages of Kitapilimwa, Luganga, Mfyome, Itagutwa, Ikengeza, Magozi, Igingilanyi, Kinywanganga, Kiwere, and Kisingha. Altitudes in both sites range from 500–1200 masl, annual rainfall is 500–600 mm, and mean annual temperature is 20–25 °C [37]. The topography in the study areas was analysed with ASTER GDEM Digital Elevation surface and little difference in elevation was found (656 and 410 m difference in IDO and KIW respectively).

### Data

We established a forest vector by supervised classification of a Landsat TM scene of 6 December 2009. A majority filter cleaned up minor forest patches outside the forest and minor gaps inside. The scene was captured at the beginning of the rainy season where there is highest separability between ground herbs and foliage of shrubs and trees. We used a very high resolution (VHR) QuickBird (QB) image for ground truthing and obtained an overall accuracy of 77.4 %, with Kappa coefficient 0.56. We compared available local GIS data, the National Reconnaissance Level Land Use and Natural Resources Mapping Project of 1997 [38], and found poor agreement with settlements in the QB image. Settlements were therefore digitized on single house level directly on VHR images from 2003 with Google Earth^TM^. The quality was visually approved with QB in KIW. We established 25 transects, each 4 km, at regular distances perpendicular to the forest edge in each site. In KID, a major vehicle road across the western part of the forest was considered as forest edge. In order to maintain the direction perpendicular to the forest edge, while respecting the systematic random sample design, all transects face south-east in area 1 and north in area 2. As the purpose of this study is to quantify forest degradation and not deforestation, all transects start at the forest edge. Above ground biomass loss was recorded in circles with radius 15 m, established at distances 500, 2000, and 3500 m along the transect. Diameter (DM) of all stumps >5 cm remaining after wood extraction was recorded. Stump DM was measured at 20 cm above ground or at height of the cut. In order to list local uses of the forest and reasons for wood extraction a qualitative pre-study was carried out. This pre-study was based on interviews with different groups of forest users and key informants including charcoal makers, fuelwood collectors, natural resource committee members, and pastoralists. Forest degradation activities in the areas include fuelwood extraction for domestic and commercial use, commercial charcoal production, poles and logs for local construction, animal grazing, sub-canopy fires and clearance for agriculture in small patches. In the field, local forest users with experience in a variety of wood harvest activities and knowledge of species participated in classification of stumps to commercial charcoal production or subsistence uses. They mainly used species, type of cut, stump DM, and location of the stumps in relation to other signs of degradation activities to decide the type of wood product associated with each stump.

### Estimation of biomass loss

**Fig. 6:**
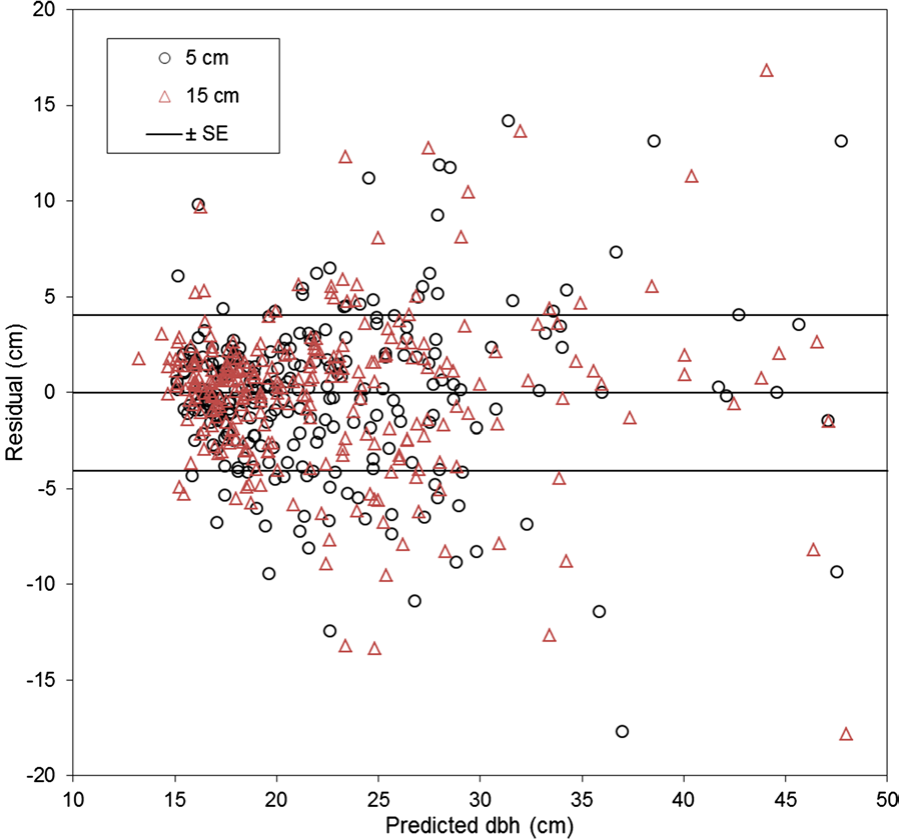
Residuals for the stem shape model. Residuals for the prediction of DBH from diameter measurements (Eq. 1). Measurement heights above ground are 5 cm (*black circle*) and 15 cm (*red triangle*). The standard error (SE) is marked by the horizontal *black lines*

Biomass loss associated with total subsistence wood extraction at each plot was quantified through measurement of diameter and height of stumps. Equations for conversion from stump diameter into diameter at breast height (DBH) have previously been established locally in Miombo woodlands [39, 40]. For this study, a local equation was established from diameter measurements on 336 reference trees in 25 circular plots of 15 m radius established in a 0.5 km grid in KIW (Eq. 1). The diameters of the trees were measured at three heights (5, 15 and 130 cm) in order to establish a conversion model that facilitate the varying heights of stump diameter measurements.

1 $$DBH = D_{s} \left( {\frac{130}{{H_{s} }}} \right)^{a} \exp \left( {b \left( {130 - H_{s} } \right)} \right),$$

where DBH is estimated tree diameter at breast height in cm, D_s_ is measured diameter of stump in cm and H_s_ is the height above ground of the D_s_ measurement in cm. Model specifications are provided in Tables 3 and 4. Accuracy of Eq. 1 was tested on the reference trees and showed diameter deviations with standard deviation 9.08 cm when estimated with DM measured at 5 cm above ground, and standard deviation 7.79 at 15 cm above ground (Fig. 6).

**Table 3:** Analysis of variance

Source	DF	Sum of squares	Mean square	Approximate F value	Pr > F
Model	2	289,095	144,548	8775.58	<0.0001
Error	532	8762.9	16.716		
Uncorrected total		534	297,858		

Analysis of variance table (ANOVA) corresponding to estimates of the stem shape model (Table 4)

**Table 4:** Parameter estimates of the stem shape model

	Approx. parameter estimate	Std error	Approximate 95 % confidence limits	
b	−0.0452	0.0173	−0.0792	−0.0113
c	−0.00108	0.000396	−0.00185	−0.00030

Parameter estimates and approximate 95 % confidence limits (by nonlinear least squares) of the stem shape model for estimation of DBH from stump diameters at 5 and 15 cm above ground. Reference data set of 336 trees

To derive biomass, an allometric equation developed for Miombo woodland in Tanzania was applied [40]:

2 $$Biomass = \exp \left( { - \, 2.959 + 2.603 \, \ln \left( {DBH} \right)} \right),$$

where Biomass is given in kg per tree including all stems and branches, and DBH is in cm.

### Data analysis

In this study, we assume that above-ground biomass loss by wood extraction in dry forest follows the distribution of a Tweedie random variable. For an exponential dispersion model (EDM), the variance of Y can be written as Var (Y) = ϕV(μ), where μ is the mean, ϕ is the dispersion, and V() is the variance function. A Tweedie distribution is an EDM where Var (μ) = ϕμ^p^. Thus, before running a generalized linear model (GLM), we need optimization to find parameters for density function *p* and dispersion ϕ. Ordinary optimization with maximum likelihood or quasi-likelihood is inadequate for establishing the Tweedie density function [26] and previously, in lack of a better approach, the parameter for density function was selected a priori with various limitations [28]. In this study, we applied optimization by use of adjusted profile likelihood, implemented in the R language and environment for statistical computing [25, 28]. The Tweedie family includes most of the important distributions commonly associated with GLM, including the Normal (p = 0), Poisson (p = 1), and Gamma (p = 2) distributions [28]. We restrict the distributions to those where the density function is 1 < p < 2. These are known as either “compound gamma” [28] or “Compound Poisson” (CP) with Poisson-Gamma random variables generated as listed below [26]:

3 $$Y = \mathop \sum \limits_{i = 1}^{T} X_{i} , T \sim Pois \left( \lambda \right), X_{i}^{iid} \sim Ga\left( {\alpha , \gamma } \right), T \bot X_{i} ,$$

where *T* is the number of plots where above-ground biomass loss is encountered and *X*_*i*_ is the measured biomass loss for the *ith* plot. The Poisson random variable is denoted by* Pois* (*λ*) with mean *λ*, and *Ga* (*α, γ*) is a Gamma random variable with mean * α* *γ* and variance *α* *γ* ^2^. When T = 0, then *Y* = 0, and when *T* > 0, the response variable Y is the sum of T i.i.d. Gamma random variables* X*_*i*_, implying that* Y*|*T* ∼* Ga* (*T* *α*,* γ*). This is why the Compound Poisson GLM has been found ideal for various studies on zero-inflated positive continuous data [26]. We applied a logarithmic link function in the GLM model, Eq. 4.

4 $$\ln \left( \mu \right) = {\alpha} + {\beta} dist + {\gamma} loc,$$

where *μ* is the expected biomass loss, *α*, *β* and *γ* are model parameters, *dist* is distance to nearest settlement and *loc* is a binary location dummy with IDO = 0 and KIW = 1. Confidence intervals were calculated using random draw Jackknife bootstrapping on the dataset and re-estimating the model 10,000 times.

Prediction models with CPGLM and confidence intervals by bootstrapping were implemented using the R environment for statistical computation and graphics. The tweedie models were computed with four different R packages; Statmod [41], Tweedie [42], CPLM [26], and fishmod [43] and results were comparable. Here we present the results using the CPLM and the fishmod packages.
